# De-implementation and substitution of clinical care processes: stakeholder perspectives on the transition to primary human papillomavirus (HPV) testing for cervical cancer screening

**DOI:** 10.1186/s43058-021-00211-z

**Published:** 2021-09-23

**Authors:** Erin E. Hahn, Corrine Munoz-Plaza, Danielle E. Altman, Chunyi Hsu, Nancy T. Cannizzaro, Quyen Ngo-Metzger, Patricia Wride, Michael K. Gould, Brian S. Mittman, Melissa Hodeib, Krishnansu S. Tewari, Lena H. Ajamian, Ramez N. Eskander, Devansu Tewari, Chun R. Chao

**Affiliations:** 1 Department of Research and Evaluation, Kaiser Permanente Southern California, 100 S. Los Robles Ave, Pasadena, CA 91101 USA; 2 Department of Health Systems Science, Kaiser Permanente Bernard J. Tyson School of Medicine, Pasadena, CA USA; 3 Center for Health Living, Kaiser Permanente Southern California, Pasadena, USA; 4 Southern California Permanente Medical Group, Pasadena, USA; 5 Department of Gynecologic Oncology, University of California Irvine, Irvine, CA USA; 6 Division of Gynecologic Oncology, Department of Obstetrics, Gynecology and Reproductive Sciences, University of California San Diego, La Jolla, CA USA

## Abstract

**Background:**

New cervical cancer screening guidelines recommend primary human papillomavirus (HPV) testing for women age 30–65 years. Healthcare organizations are preparing to de-implement the previous recommended strategies of Pap testing or co-testing (Pap plus HPV test) and substitute primary HPV testing. However, there may be significant challenges to the replacement of this entrenched clinical practice, even with an evidence-based substitution. We sought to identify stakeholder-perceived barriers and facilitators to this substitution within a large healthcare system, Kaiser Permanente Southern California.

**Methods:**

We conducted semi-structured qualitative interviews with clinician, administrative, and patient stakeholders regarding (a) acceptability and feasibility of the planned substitution; (b) perceptions of barriers and facilitators, with an emphasis on those related to the de-implementation/implementation cycle of substitution; and (c) perceived readiness to change. Our interview guide was informed by the Consolidated Framework for Implementation Research (CFIR). Using a team coding approach, we developed an initial coding structure refined during iterative analysis; the data were subsequently organized thematically into domains, key themes, and sub-themes using thematic analysis, followed by framework analysis informed by CFIR.

**Results:**

We conducted 23 interviews: 5 patient and 18 clinical/administrative. Clinicians perceived that patients feel more tests equals better care, and clinicians and patients expressed fear of missed cancers (“…it’ll be more challenging convincing the patient that only one test is…good enough to detect cancer.”). Patients perceived practice changes resulting in “less care” are driven by the desire to cut costs*.* In contrast, clinicians/administrators viewed changing from two tests to one as acceptable and a workflow efficiency (“…It’s very easy and half the work.”). Stakeholder*-*recommended strategies included focusing on the increased efficacy of primary HPV testing and developing clinician talking points incorporating national guidelines to assuage “cost-cutting” fears.

**Conclusions:**

Substitution to replace an entrenched clinical practice is complex. Leveraging available facilitators is key to ease the process for clinical and administrative stakeholders—e.g., emphasizing the efficiency of going from two tests to one. Identifying and addressing clinician and patient fears regarding cost-cutting and perceived poorer quality of care is critical for substitution. Multicomponent and multilevel strategies for engagement and education will be required.

**Trial registration:**

ClinicalTrials.gov, #NCT04371887

**Supplementary Information:**

The online version contains supplementary material available at 10.1186/s43058-021-00211-z.

Contributions to the literature

- There is growing interest in clinical practice de-implementation and substitution research, but relatively few real-life examples have been studied.
- Interviews with clinical, administrative, and patient stakeholders in a health system transitioning to a new cervical cancer screening process identified several barriers and facilitators which are similar to traditional implementation efforts, including the importance of leadership and local champions and the acceptability and feasibility of the change.
- Additionally, interviews revealed several unique facets of de-implementation/substitution to address, including concerns about cost-cutting and reduced quality of care.
- These findings will inform our evolving understanding of how to best facilitate clinical practice change.

## Background

National organizations including the United States Preventive Services Task Force (USPSTF) are recommending a relatively new approach for cervical cancer screening for women aged 30–65 years known as *primary human papillomavirus* (*HPV*) testing [1]. The use of primary HPV testing is also endorsed by the American College of Obstetrics and Gynecology (ACOG) [2]. Studies suggest primary HPV testing is superior to cytology alone (Papanicolaou testing (Pap)) [3] and is as effective as co-testing (Pap plus HPV test) [4–7]. As a result, US healthcare organizations are preparing to de-implement the previous recommended strategies of co-testing or Pap alone and initiate primary HPV testing. While few other countries have fully implemented primary HPV testing, it is strongly recommended in European guidelines and many countries are working towards full implementation [8]. Both the Netherlands and Turkey have transitioned to primary HPV testing, as well as Australia [9, 10]. Based on these experiences and modeling studies, the transition to primary HPV testing is expected to result in improved cost-effectiveness and simplified clinical care processes for those converting from co-testing to primary HPV testing (e.g., one sample taken instead of two at point of care) [11, 12]; primary HPV testing may also lead to better patient outcomes and will align with the new guidelines and associated quality metrics. These may be powerful facilitators for change. However, evidence is lacking regarding effective strategies for this type of dual-practice change incorporating de-implementation plus substitution with a new recommended practice.

Substitution is a relatively new concept and research focus in the implementation and improvement science fields. Recent scholarly and clinical efforts focusing on de-implementation have concentrated heavily on the cessation of contraindicated practices and/or overuse of practices that cause more harm than benefit [13–17]. In the field of cancer prevention and control, overuse is common and includes overuse of screening services, overtreatment, and overtesting during surveillance [18–22]. A recent editorial on de-implementation in cancer care distinguished between four types of cancer-related practices to be considered for de-implementation: ineffective, contradicted, mixed, and untested [18]. In general, these practices do not have an available substitute, and research and quality improvement efforts have focused on discontinuation or reduction in frequency. For example, the change in the USPSTF recommended initiation of breast cancer screening for low-to-average risk women from age 40 to age 50 represents a discontinuation of screening without substitute; similarly, the American Society of Clinical Oncology recommends that asymptomatic post-treatment breast cancer patients do not need surveillance imaging or laboratory tests. An example of the reduction in frequency is the evolving guideline recommendation to extend the intervals between cervical cancer screenings for low-to-average risk women. Many current de-implementation efforts in cancer care and other clinical disciplines stem from the Choosing Wisely campaign from the American Board of Internal Medicine, which focuses on clinical practices that could be reduced or completely ceased across a variety of clinical domains [13, 23–26].

In contrast to de-implementation, substitution has been defined as the promotion of one or more alternatives to an ineffective or less effective practice, in which the substitutes replace or displace the previous practice [27–30]. Ideally, to ease cognitive pressures and potentially simplify change management strategies, a substitution will have a *related* replacement that is a similar, closely related but more effective intervention [14, 28]. Substitution can be seen as “coupled” implementation and de-implementation [28, 29] and as a potential strategy to facilitate practice change. While implementation of novel practices is certainly challenging, de-implementation of established, routine practices is also challenging [15, 24, 31, 32]. It is possible that a coupled approach, if available, could be more favorably received by clinicians and health systems [14, 28, 29]. A coupled strategy would require a multicomponent intervention that encompasses both de-implementation and implementation goals, requiring strategies to address cessation of the existing practice and replacement with the newer, more effective practice [28].

Within the USA, the decision to transition to primary HPV screening is mainly left to individual health systems rather than implemented on a national or state level. The decision of health insurers (governmental and private) regarding coverage may influence uptake. Kaiser Permanente Southern California (KPSC), a large integrated health system (hospitals, medical groups, and insurance plan) transitioned from HPV co-testing to primary HPV testing in July 2020 in response to the updated USPSTF guidelines. As part of a multi-year study evaluating implementation strategies to facilitate HPV primary testing, we conducted a series of semi-structured qualitative interviews with KPSC members, clinicians, and administrators. Interviews were conducted prior to the practice change and any related educational efforts (e.g., clinician webinars) to gain insight into perceived barriers and recommendations for change. The overarching goal of the main study is to compare implementation strategies (tailored versus centralized strategies) to facilitate uptake of primary HPV testing. Interviews with clinicians and administrators elicited their views on (a) the acceptability and feasibility of the planned substitution; (b) their perceptions of potential barriers and facilitators, with an emphasis on those related to the de-implementation/implementation cycle of substitution; and (c) perceived readiness to change to primary HPV screening. We also explored potential strategies or recommendations to overcome potential multilevel challenges (patient-, clinician-, and system-level). Patient interviews focused on overall reactions to the proposed change and attitudes regarding this new approach to cervical cancer screening.

## Methods

### Study design

We conducted a qualitative study using semi-structured interviews; our findings are reported using the criteria for reporting qualitative research (COREQ). The COREQ checklist can be found in Additional file 1.

### Study setting and participants

KPSC is an integrated delivery system serving over 4.4 million members. Members are racially, ethnically, and socioeconomically diverse and broadly representative of the underlying Southern California population [33]. We conducted interviews with clinicians and administrators between April and November 2019 and with patients from July 2019 to November 2019. The following types of clinical and administrative stakeholder participants were invited to participate: (1) Family Medicine, Internal Medicine, and Obstetrician-Gynecology physicians; (2) department administrators (DAs) and assistant department administrators (ADAs); and (3) nurses including medical assistants (MAs) and licensed vocational nurses (LVNs). For patients, we invited individuals who were female, aged 30–65 years, who received cervical cancer screening within the last 3 months and were English speakers. We obtained verbal consent from all participants, which was approved by the KPSC Institutional Review Board [IRB #12015], and a written informed consent from the patients for audiotaping and transcribing.

### Interview guide and conceptual model

Interviews with both clinical/administrative and patient participants were facilitated using semi-structured interview guides. The guides included open-ended questions based on central research questions and probing questions, which allowed for deep exploration of participant responses and probes for emergent themes. Our iterative interview guide development was informed by the Consolidated Framework for Implementation Research (CFIR) [34]. The CFIR is widely recognized to provide a comprehensive overview of relevant constructs applicable to implementation research and clinical practice change [35]. We developed potential interview questions based on the HPV testing and implementation science literature and mapped the question domains to relevant CFIR constructs (see Additional file 1). We convened our study’s patient advisory group (3 KPSC patient members, 1 non-KPSC patient) to inform the development of our interview guides (i.e., *Were we missing any key questions? Did we need to revise the interview question flow?*); this group also provided input on the development of a patient-, clinician-, and system-level list of potential implementation barriers—designed with our clinical stakeholder group—to be used during the interviews with clinical and administrative participants (see Additional file 2). The interview guide was further discussed and refined by the study team after the initial interviews.

Domains in the clinician/administrative guide included (a) awareness, knowledge, and beliefs related to the practice change; (b) potential barriers to de-implementing current co-testing practices and substituting primary HPV screening; (c) perceived patient reactions to the substitution and related needs and resources; (d) site-specific culture and contextual issues with potential to impact substitution (i.e., culture, leadership, readiness-for-change); (e) clinician self-efficacy; (f) recommendations to facilitate change; and (g) champions or key leadership available at their site. Topics covered during patient interviews included (a) perceptions of guidelines and medical evidence; (b) facilitators and barriers to de-implementation and substitution, and overall acceptance of primary HPV testing; (c) identification of patient sub-populations experiencing potentially greater barriers to acceptance of primary HPV testing; and (d) strategies for increasing acceptance of this substitution as well as prioritization of strategies.

### Recruitment

To attain theoretical data saturation, we planned 20–25 total interviews. We emailed invitations and three reminders to 238 physicians, 82 administrators, and 400 patients, assuming a 10–15% response rate based on our prior experience in conducting qualitative research with these stakeholder groups [21, 36, 37]. No prior relationships existed between the interviewers (CMP, DA) and participants prior to study commencement. Participants were informed about the interviewers’ role as qualitative researchers on the study team, who had an interest in improving cervical cancer screening practices.

### Interview procedures

We conducted one-time individual semi-structured interviews. Clinician and administrator interviews were conducted in person or over the phone in a private location within the medical center by CMP, a female qualitative researcher with over 25 years of experience, and DEA, a female qualitative researcher with over 10 years of experience. Patient interviews were conducted by phone to minimize travel burden. Interviews lasted approximately 30–60 min and were recorded and transcribed verbatim into written transcripts as preparation for coding and analysis using NVivo qualitative analytical software (© QSR International 2020). We halted further interviews once we were confident that we reached thematic saturation with our interview sample whereby we were no longer eliciting new pertinent information or themes from additional interviews [38–41]. Transcription was conducted by an institutionally approved vendor. We collected age, gender, race/ethnicity, and profession data from KPSC’s electronic health records and/or participant self-report.

### Coding and analysis

We conducted a two-step analysis with initial thematic analysis, followed by framework analysis guided by CFIR. This two-step process allows for an initial free-form analysis with exploration of convergent and divergent findings, followed by a mapping process to the overarching CFIR framework without the possibility of missing divergent/discordant findings. We developed an initial coding tree structure which we refined during the analysis process. To help ensure rigor and transparency, all coding development steps were tracked and reported in a codebook [42]. Consensus on the coding structure was achieved through cyclical testing of structure development by three members of the research team using a team coding approach to review and resolve discrepancies. We identified a lead coder (CMP) and two secondary coders (EEH and CH). Coders independently coded a random sample of the transcripts (*n* = 4), then met repeatedly to compare coding applications, discuss analytic insights and annotations, and identify and resolve discrepancies. Once consensus was achieved and discrepancies resolved, the codebook was finalized and the lead coder continued to code the remaining transcripts. Once coding was complete, the team met as needed to determine the hierarchy of themes and produced an aggregated summary of the findings categorized into domains, themes, and sub-themes. We categorized recommendations and suggestions to address barriers with the goals of (1) informing the design of the process-change activities across the organization and (2) understanding cross-cutting themes across stakeholder roles. Transcripts and data analysis were not returned to participants. While brief notes were used in post-interview summaries shared with the study team, traditional field notes were not included as part of the formal analysis.

## Results

### Sample characteristics

Of the 238 clinician invitations, 26 (11%) responded yes to the interview request and 14 completed interviews. For administrators, 11 (14%) of the invited 82 responded yes, and 4 completed interviews. For patients, 10 (3%) of the invited 400 responded yes, and 5 completed interviews. We conducted a total of 23 interviews, *n* = 18 clinician/administrator and *n* = 5 patient. Table 1 provides a breakdown of the stakeholder roles and characteristics.

**Table 1:** Qualitative interview participant characteristics

Stakeholder role		Clinician	Administrator	Members
*N*		14	4	5
Mean age		44.8	Range only, 30–39 (2); 40–49 (2)	43.2
Gender	Female	14	3	5
	Male	0	1	0
Race/ethnicity	Asian	5	1^a^	1
	Hispanic	4	2^a^	0
	White	4	1^a^	3
	Black	1	0^a^	1
Specialty/department	Family med	7	1	N/A
	Internal med	2	0	
	Ob/gyn	3	3	
Clinical role	Physician	9	N/A	N/A
	Nurse	5		

^a^Administrator race/ethnicity derived from the surname

### Thematic findings

#### Clinician resistance to substitution

Participants perceived that some clinicians, especially in primary care, may be more resistant to this practice change due to the dual challenges of the substitution, encompassing both de-implementation (co-testing) and implementation (primary HPV testing). One physician referenced colleagues who are still struggling to adapt to older guideline changes with regard to cervical cancer screening frequency:

I’m going to be very honest with you…there are still some providers who have that old practice of wanting to do [Pap tests] every year. So…CDC guidelines are the three years. We’ll just use the three years as the minimum. There are still providers who insist on doing it every year, so we have…the over testing.

The clinical stakeholders most reluctant to accept the practice change may be members of the nursing team who have their own attachment to the Pap test/co-testing based on their personal screening experience and knowledge. A nurse worried, “I didn’t know they were going to eliminate Pap, because I’ve seen Pap [be] abnormal in the past and not be HPV ...I always worry are we making sure we are capturing everything?,” while a family medicine physician perceived that “…most of our nursing staff are women, so they themselves might have some personal bias for or against Pap changes. I think you would have to make sure that there is buy in….”

Furthermore, participants shared concerns that patient resistance and/or confusion will require clinicians to engage in longer conversations with “anxious” women:

Patients who just want to come in and patients who are unfamiliar with it will want specifically a Pap. That, again, takes time now from the physicians to convince or inform the patient, you don’t need that. You don’t need X, Y, and Z. Here’s the research, here’s the reasons why. And having those kind of like continued conversations by…patients [asking] ‘Why didn’t I get a…Pap smear?’

Highlighting that not all clinicians have the skills to communicate information about practice changes to their patients effectively, participants suggested “…it’s also [about] educating the physician” and “…[making] sure our physicians are…well-informed about how we communicate this to patients on a mass level, so that they don’t come in expecting…something else….”

#### Patient attachment to the Pap

Women’s attachment to the Pap (especially older women) was identified across participants as a critical challenge to the substitution. Clinicians and department administrators noted that women do not always respond to population-level statistics, which can feel impersonal, citing that many patients still demand a yearly Pap instead of the recommended interval of co-testing regardless of how much the evidence is explained to them (“…still, some of my older patients, they’re like, ‘I don’t feel comfortable not having a Pap smear every year.’”). One physician who has a high proportion of Hispanic patients noticed that they often travel to Mexico to get yearly Pap tests, while another fears “…patients will continue to request certain [tests] because we already currently have…some patients who want over screening.” Patients raised the same concerns. One woman said that it was a good thing she learned about this potential practice change in advance through her participation in the interview, because “…If I didn’t know I’d be surprised and I’d be distrustful, why did you take my [Pap] test away?”.

#### Fear of missed cancers/more care is better care

While many participants framed it as an attachment to the Pap test specifically, a deeper analysis suggests the fundamental basis of the concern about the substitution of primary HPV testing in place of co-testing centers around the fear of potentially missing cancer. A patient shared her concerns: “…just to make sure nothing is going to be missed, just assuring patients that this test is going to be able to find -- detect X, Y, and Z just like the Pap….” Some clinicians (particularly nurses) also expressed concern about missed cancers:

…I’ve seen Pap [be] abnormal in the past and not be HPV…Trust me, if you’re down there, what’s the difference if you take one more scraping of something? I don’t understand why they would discontinue one, take one type of abnormal cell and not go, oh, because I’ve seen them both come back abnormal at different times. I don’t understand the logic. I guess somebody would have to explain it to me, we want to eliminate it because of this amount of chance, or this percentage. But, again, if it’s your family member, do you want to be that percentage of only five percent will end up with this particular kind of cancer?

Interviews with both patients and clinicians strongly suggest that some patients hold fast to the idea that the more tests and procedures they get, the better the quality of their care. The challenge from the clinician perspective is “…convincing the patient that only one test is needed and good enough to prevent and to help detect cancer.”

Patient data suggest this concern is justified, as they question if “The standard of care right now [for women’s health] is kind of going down because the research being done is saying, ‘We don't need to do these things as often…’” and “…if we're getting rid of the regular Pap smear - is this HPV test going to be screening for all the rest of it that the regular Pap smear also screened for?” Clinicians shared numerous stories of difficult conversations with patients about changing practices to get them to understand the concept of de-implementation (e.g., the cessation of healthcare services that do not provide benefit) and that receipt of more services does not always result in better health outcomes and may even lead to harms, including unnecessary tests and procedures.

#### Cost-cutting and competition

Clinicians, administrators, and patients repeatedly mentioned a perception among patients (and some clinicians) that de-implementation and/or substitution efforts resulting in fewer tests and services are fundamentally driven by the desire to cut costs and/or make procedures more convenient for the clinical team and healthcare system, rather than being driven by quality and new evidence. Echoing the sentiment of many participants, a family medicine physician lamented:

…[you have to] sit and educate the patient…Because it’s always if you don’t explain it adequately that the patient somehow feels they’re being slighted, or they’re not being provided care. That we’re somehow, ‘Oh, we’re doing this to save money.’ It’s like, no, we’re not, we’re doing this because this is what evidence is seeing.

Some patients have certain expectations because of the co-pays required for their visits and procedures: “…there are patients that say, ‘I always feel like [KPSC]…is just cutting stuff out and they’re already taking my money…‘Why aren’t they just doing the Pap every year?’” In addition, clinicians shared their concerns about external perceptions if their system moves to a practice that is different from local competitors because such a shift may influence patient perceptions regarding the system’s motivation to initiate the change (e.g., will it fuel patients’ suspicions about organizational motives, thereby hurting the organization’s reputation and standing in the community?). Noting that patients come into Kaiser Permanente from other healthcare systems, or hear about the services and procedures their friends and/or family receive from other health organizations, one DA (ob-gyn) said:

Whatever is the community standard…it is more difficult for us to now say we are going to do this versus [other healthcare systems] who are in our backyards, that they will continue to do PAP smears with HPV and then we’re the odd people out…[patients then say] ‘Okay, well I get more tests [from other systems] and that is better care.’

Even some clinicians questioned the motivation for the change (“The most important thing is quality, second thing is cost. I just want to make sure we are not putting cost above quality.”) along with patients (“Yeah, I mean, I’m definitely not just this healthcare is a conspiracy [patient] or anything like that, but...I know that there’s corners cut …I would be wondering if that's what’s going on here, too.”)*.*

#### Impact on access and workload

Clinical and administrative participants questioned whether the change to primary HPV testing and/or the new follow-up algorithm will result in a higher volume of patient questions and inquiries, either via increased email messaging, telephone calls, or lengthier visits (“Yeah, this is always a worry for primary care, that we’re going to get more questions or emails or it’s going to increase our indirect work…”)*.* These participants also raised concerns as to whether the change will exacerbate pre-existing access problems in primary care and/or ob-gyn, “…Because if that’s a thing that we have to have patients come back [more]? I don’t think we have the capacity…That’s always the biggest problem for us, is access.”

While a minor theme, clinicians expressed concerns that there may be a steep learning curve to understand the new follow-up algorithm for abnormal primary HPV results. Others felt this challenge could be easily addressed with clear education and communication, “…a lot of the people here are just used to doing the cytology [Pap] and HPV. So, they know the protocol. But then, it’s a little bit different with the primary HPV but…you’ve just got to read a little bit about it.” However, an ob/gyn physician expressed a bit more concern about the learning curve for her colleagues in primary care:

I’m wondering if they’re going to completely understand in family medicine. And are they going to be able to explain to their patients correctly without saying, ‘Well, you know what? Just go be seen in Ob/Gyn.’

### Implementation facilitators

#### Substitution with replacement: feasible and acceptable

For many clinicians and administrators, this substitution with replacement is viewed as relatively minor and an overall workflow efficiency. One DA commented, *“*In my mind, as an operational leader, what satisfies the patient’s needs, provides the same level of care, and also makes the work of my staff and my team easier, I’m all for.” Participants in both clinical and administrative roles reiterated that they believe the change will make specimen collection easier (“I don’t think it’s going to be much of a challenge or a barrier to get the workflow started…. It’s a very easy and half the work.”) and nurses will appreciate having less to do to prepare the specimen collection trays (“Okay, one less thing we have to set up. That’s great.”). However, while a primary care physician also found the proposed practice change to be acceptable and feasible (“…if we’re just going to switch to that because we know [HPV] is the cause, it seems pretty reasonable.”), she added an important caveat mentioned by other participants as well, “I just think as long as we educate both the physicians and then the patients secondarily, it should be okay.” Another DA highlighted that a workflow change that is viewed as an efficiency can be leveraged for buy-in: “If it’s something that…reduces the workload of the providers or the staff, then generally it’s going to be accepted and adopted quicker.” In addition, based on the current evidence, the change is perceived as likely to result in fewer unnecessary and invasive tests over time. (*“*Maybe doing less invasive procedures. You can avoid that.”)

#### Evidence to support substitution

Clinical and administrative participants reported having an expected threshold for the level of evidence they believe will be necessary to secure buy-in for the planned practice change to primary HPV testing (“With the support of the education, it will not be difficult.”). The level of evidence desired is described as basic, brief, and from respected external specialty associations and organizations (i.e., ACOG and the USPSTF).

#### Medical center culture

Descriptions of high functioning clinic culture with adaptability regarding de-implementation, implementation, and/or substitution were linked in particular to strong leadership by the DAs, because clinicians often look to them to set a tone of positivity in the clinic and lead practice change. One DA explained, “You have to build relationships with your staff. Your staff needs to trust you. And when [when] that time comes [for change], they are accommodating to you.” In addition, teams often described having facilitated reasonably smooth implementation of similar practice changes (“…we’re a pretty supportive group…It’s pretty easy. We haven’t had any major issues when we’ve had changes…”). Finally, most clinics already have identified “champions” or clinicians with subject expertise that can be leveraged (i.e., cervical dysplasia champions, primary care physicians with an interest in women’s health issues, etc.):

…we have a lot of that here, and each physician sometimes has special interests or things that they’re good at and…everybody knows, I’m the women’s health person…And then we have another guy that’s really into communication with patients and difficult patient encounters…we try to…influence each other, and we know who to go to when we have questions.

### Alignment with CFIR

Viewing our findings through the lens of the CFIR framework is informative. Guided by the CFIR *Intervention Characteristics* domain, we asked stakeholders to reflect on the strength, complexity, and design of the planned practice change intervention. We discovered that stakeholder perceptions of *acceptability* (Intervention Characteristics) are high in terms of the workflow impact on clinical teams, suggesting a key facilitator that can be leveraged for successful substitution. Perceptions that the change may result in fewer unnecessary and invasive tests per the updated evidence in the new guideline fed into opinions about the acceptability of the practice change as well. However, participants repeatedly pointed out that the acceptability from the patient perspective may be lower, at least for an important minority of patients, and is a barrier that should be anticipated and met proactively. Under the CFIR domain *Individual Characteristics*, physician confidence was also reportedly high in terms of their belief that they and their colleagues could easily transition to the new cervical screening practice. Suggested implementation facilitators related to positive team culture, strong DA leadership, successful implementation of previous workflow changes, and an existing network of subject matter experts and physician champions fall into the CFIR domain *Process & Inner Setting* and also indicate a reasonably high feasibility for implementation success in the practice setting.

System-level barriers, including the impact on access and workload, as well as uncertainty about follow-up testing algorithms and patient transitions between primary care and ob-gyn fall into the CFIR domain *Intervention Characteristics & Outer Setting.* Potential clinician resistance and apprehension about the substitution falls into *Characteristic of Individuals* and should be anticipated and addressed proactively. Similarly, identified patient concerns (e.g., what happened to the PAP test?), fears (e.g., missed cancers), and potential for resistance align best with CFIR’s *Patient Needs & Resources* domain and were perceived by stakeholders as the most critical barriers to address.

## Discussion

Overall, our results demonstrate the complex nature of substitution with replacement and the de-implementation/implementation cycle of practice change. On one hand, clinicians and administrators indicated that this change in cervical cancer screening is viewed as relatively minor and a workflow efficiency. De-implementing a two-test process (co-testing with both HPV and Pap) and offering an acceptable, simpler substitution option (primary HPV test only) is a strong facilitator for this practice change. Participants recommended leveraging this perception—e.g., the substitution will make staff jobs easier, not harder—to secure buy-in from clinicians and administrators in advance of the practice change rollout. On the other hand, patients and some clinicians expressed strong fears of potential missed cancers, questioned the reason for the change, and perceived that de-implementing Pap testing was driven by cost-cutting and would result in lower value of care for women. Relatedly, we observed a common belief that more care equals better quality of care. Fears regarding cost-cutting and “more is better” are unique facets of de-implementation and/or substitution that will need to be addressed to facilitate successful practice change. This may be of particular concern in the USA, which has a competitive healthcare market [43] where patients and health insurance purchasers may select and change clinicians and/or health systems based on costs, coverage, and preferences. Typically, implementation efforts and strategies focus on the adoption of novel technologies or treatments, rather than de-implementation of low-value services [44–47]; the addition of domains specific to patient and clinician perceptions of cost-cutting, healthcare competition, and more tests and services equaling better care could strengthen existing practice change frameworks [48–50]. Participants suggested clinicians should be sufficiently prepared to have difficult conversations with anxious patients about the substitution. Additionally, participants recommended the development of clinician scripts and talking points and/or physician training on how to effectively discuss evidence and high-value care with patients (e.g., introducing the concept of de-implementation in relation to the reality of shifting medical evidence) [27, 32].

Participants offered other suggestions for multicomponent strategies to overcome potential system-, clinician-, and patient-level barriers. These suggestions include developing and delivering brief, concise clinician- and patient-facing education materials and resources to secure buy-in from clinicians and assuage patient fears that primary HPV testing will leave them vulnerable to missed cancers. In both cases, participants suggest using multiple channels for education both prior to and during the substitution processes. In the case of clinicians, specific suggestions included departmental announcements via email and at meetings (from departmental Chiefs of Service and DAs), in-service trainings from internal/external subject experts or “champions,” stories of positive patient impact, and independent online learning modules. For patients, specific suggestions for resource development included posters in exam rooms and communal areas within medical centers, videos, media/social media campaigns, information on the patient portal, personalized letters, and targeted reminders tied to the visit for the procedure, with messaging focused on the increased sensitivity of primary HPV screening for detecting and preventing cervical cancer and incorporating national guidelines to assuage the “more care is better” and “cost-cutting” fears. Specific recommendations to address identified barriers are presented in Table 2 and summarized briefly below.

**Table 2:** Participant suggestions for cervical cancer screening practice change

**Suggestions to address patient barriers**					
**Barrier**	**Suggested solution**	**Details/examples**	**Representative quotes**		
**Patient concerns that the practice change is actually “less care” due to one test instead of two; perception of decreased quality of care**	Clinician script and/or training on discussing evidence and high-value care	Accompany script with external resources (e.g., CDC, NIH) to explain the change—addresses concerns that KP or other health system is cutting corners or not providing the best care Explain to patients that medical evidence is constantly changing Ex script: *At KP, we want to provide you the highest quality, most evidence-based care. You can see from these national resources that primary HPV testing is the best possible choice for patients like you. While I can’t speak to what is going on in other systems, I can tell you that we want to do the best possible thing for you.*	*To make awareness of the patients to know that the test is being changed for the better and for more accurate and speedy results. Any kind of change you want it to be for the better and not just switching over because we think we should. We want a reason for it and the reason has to be better results. HPV_PT_001, Patient*	*If you go look at some of the research papers…They talk in more medical or science-based terminology that's not always something everybody is going to understand. I think the summaries are better than the full-on research paper…So, I would keep it simple but enough that the person who needs to be tested would trust…Because individually, I think it needs to be something that shows them why you're making that change and what the science is. HPV_PT_002, Patient*	*Yeah, I’m kind of concerned because what if I go in for my Pap, and I am told I will no longer be getting a Pap, and let’s say I’m not -- I don’t have HPV, and they said, okay, you’re free to go, I’d be concerned because I’d normally get a Pap, and I want to know what would have been said if I got a Pap. So what I want to know is will the HPV primary testing alone -- will that catch everything that would have been caught if I had a Pap? I would like to know. HPV_PT_005, Patient*
**Patient knowledge; additional resistance to loss of PAP test, the basis of which is fundamentally the fear of “missing cancer”**	Develop a wide range of patient-facing resources for distribution both *prior* to and *during* rollout. The theme of missing cancer is the primary concern related by patients (and some providers)—*suggests the importance of addressing this fear first and foremost in ALL patient-facing materials*—i.e., “99.7% of cervical cancer is caused by HPV.”	Brochures, flyers, other “one pagers” that address: Why the change? What are the benefits? What are the potential side effects and/or risks? Other types of educational resources suggested: Posters in exam rooms and communal areas within medical centers Videos (waiting rooms, EMMI videos) Media/social media campaigns Patient portal (kp.org) Personalized letters before rollout Targeted reminder tied to the visit for the procedure	*Have a one-page document with bullet points that they can give. Sometimes you’re listening to your doctor and then you go home and say, “What?” I love taking home little pamphlets. You guys are really great at doing your infographics. You feel like at least you got something that you can refer to. Especially if you were able to accept it from the doctor but you went home and couldn’t remember any of it, you’ve got this little piece of paper that reminds you. So, I like that. It doesn’t have to be a paper, although that’s probably best because not everybody has access to online. I thought they did but I just found out they’re not. I’m surprised. HPV_PT_002, Patient*	**Interviewer***: Out of the ways you get contacted, either by email, phone or specific oPAP messaging, what’s your preferred method? What resonates with you the best?* **Respondent***: It’s hit and miss for me. They’re all three different honestly. Sometimes one is better than the other. I think my phone messaging is good. I like text messaging for certain things. For newsletter information stuff, I’d prefer that through email. HPV_PT_001, Patient*	*Kaiser is unique in the sense that every single one of your patients has to have a primary care physician. I specifically go to a gynecologist, but I know that any of the primary care physicians can perform a Pap smear. I think you’ve got the database there. You’ve got all the optics for all the women. You could start as simple as sending an email or note through the system from the doctor. You know who all of your women patients are. You know what their ages are. You could just announce there’s been a change. The next time you come in or if you have further questions, speak to your physician or gynecologist. Then have the doctors talk to you when you are in front of them. HPV_PT_003, Patient*
**Patient expectations for physician-patient communication about the switch to HPV primary testing**	Clinically, this change may be perceived as simple by the provider and administrative stakeholders, but patient perceptions and concerns may be more than anticipated—*they will likely expect/request proactive conversations with their doctors* Skipping this step in communication will likely lead to greater patient confusion and mistrust of the reasons for the change (i.e., KP is just trying to save money/increase convenience)	Physician and nurse teams need to be prepared with clear talking points and “scripts” to address this change with patients “early and often”: Encourage clinicians to broach the topic of the practice change with patients at visits unrelated to a screening visit to better prepare patients for the change	*A basic standard of care that I expect is to be given a guideline or a big picture of something. But I think they’re also responsible for working alongside you as well. A guideline isn’t the end-all-be-all. I think they’re also supposed to work alongside you and see what works best for you. HPV_PT_003, Patient*	*Tell me why what your change is better. “This is why we’re doing it.” Just by answering their question. Whether they like your answer or not, you’ve done your best. HPV_PT_001, Patient* *I think just for this, it’s just a big educational piece with patients, just letting them know it is okay, we are doing the right thing here for you…As long as they explain why they think it’s a better test and that we don’t need that Pap…So, I think that will maybe be a little education as to why that’s safe and okay. HPV_001, Family Medicine Physician*	*…just bringing it up and asking patients if they have questions…so making sure they say, “You know, I want to take a minute to talk about this screening or whatever. What questions do you have? What concerns do you have?” Just that transparency and bringing it up and keeping us aware that there has a been a change or there might be a change or whatever. HPV_PT_007, Patient*
**Suggestions to address clinician and administrative stakeholder barriers**					
**Barrier**	**Suggested solution**	**Details**	**Representative quotes**		
**Provider resistance to change**	Improve chance of buy-in by presenting a brief summary (e.g., fact sheets) of the evidence *prior* to the rollout: Providers expect evidence to include: Rationale for the practice change Underscore evidence comes from a reputable external source (i.e., ACOG, USPSTF, etc.) Assuage concerns about “missed cancers”	Provide information in multiple formats: Departmental announcements via email and at meetings (from both Chiefs and DAs) In-service trainings from internal/external subject experts or “champions” Include stories of positive patient impact Independent learning modules on KP Learn Scripts or talking points to improve patient-provider communication on the subject	*Like, there’s people that are more visual, there’s people that need to read it or need to see a demonstration…hit every learning angle, I guess, if that’s what you want to call it. HPV_006, Ob-Gyn LVN* *…if the provider is not sold on it…it would be hard for them to convince a very anxious, nervous patient that this would be the right thing to do. HPV_022, Family Medicine Physician* *Definitely a FAQs sheet. Doctors don’t like to read a whole lot of things like that...They just want the points…show me why and how… HPV_012, Family Medicine Department Administrator*	*Sometimes, at the family department meetings or the OB/GYN meeting, they would probably give out some information on that, or at the offsite [meeting], you’ll hit pretty much all the physicians there, specialists and primary docs. Emails, of course…and they just give us a little handout saying, “These are the new guidelines.”…So, it’s always good to get it from multiple sources, because I think one is not enough. HPV_001, Family Medicine Physician*	*It’s typically sort of multifactorial…usually, we’ve got visual, whether it’s flyers, the handouts, documentation, examples…demonstrate that…we’re not just throwing this out and saying, “Oh, now we’re doing this, now we’re doing that” But we’re saying, “Okay, here’s the change, here’s the benefits of the change, here’s the reasons why we’re doing the change. Here’s what you need, your part for participating, implementing this change.” HPV_003, Ob-Gyn Assistant Department Administrator*
**Provider learning curve**	Address and satisfy questions about changes: Concerns about the follow-up algorithm in particular for primary care physicians Ease concerns that the workflow change may result in increased appointments and/or access issues	Specify the new FU algorithm clearly (a simple one-page flowsheet) Bring in someone from gynecology to do a presentation Utilize champions and DAs to train physician-nurse teams before rollout/reinforce during transition For the workflow change, provide the nurse team with: Practical manual (e.g., visual aid for tray change)	*…the first [barrier], which I already mentioned to you. But – inability to perform proper follow-up in three months for women of positive results. HPV_004, Ob-Gyn LVN*	*A staff meeting and literature….If I still had questions, I would definitely call our case manager – dysplasia case manager….they would be up to date. HPV_004, Ob-Gyn LVN*	*People are very visual. You put pictures of one tube versus two tubes. So, have one tube with a circle and then two tubes you would X out…everybody has a different way of doing it so whenever I work with different MA-LVNs I sometimes have to teach them how to do it and that is a burden for physicians because it should be a natural automatic standardized information. HPV_015, Family Medicine Physician*
**Potentially most challenging provider to garner “buy-in” from for the change**	Do not overlook nurses and/or female providers who may view this change through a dual lens—as both a clinician and a female patient: As stakeholders, they may have heightened resistance to this practice change	They need clear/concise evidence; may be the most challenging to persuade, so provide evidence that speaks to the “why” as well and address their concerns head on about potential for “missed cancers” under new testing protocol Do not forget about float pool of nurses—they often miss valuable trainings	*…sometimes, they just said, “Oh, well. This is what we're implementing.”…We want to know why – why is this change happening? Are we losing anything? Are we gaining something from it?...[also]…is it ACOG-approved…Or is this just Kaiser changing it just because. HPV_004, Ob-Gyn LVN*	*For the nurses, they just want to understand why this happening and what the potential impact for them and the patients are. HPV_011, Ob-Gyn Department Administrator*	*They sometimes have floating nurses [and] there are part-time nurses, and then there are nurses who normally are on the phone, but then they end up stepping in, and they didn't get trained, and they don’t know what they're doing. So, you want to [capture] everybody. HPV_022, Family Medicine Physician*

### Barrier: patient perceptions of “lower quality” care

#### Suggested solution(s)

Create clinician-targeted communication scripts and/or training on how to effectively discuss the practice change and the concept of high-value care with patients.

Accompany the script with external resources from national organizations with credibility and standing (ACOG, USPSTF) to explain the change; this addresses concerns that health systems are cutting corners or not providing the best care.

### Barrier: patient resistance to Pap de-implementation

#### Suggested solution(s)

Recommendations included developing a wide range of patient-facing resources for distribution both prior to and during the practice change implementation. A key focus should be assuaging patient concerns about “missed cancers” with current evidence of primary HPV testing efficacy and explaining concepts of guideline-recommended de-implementation of unnecessary tests and/or procedures.

### Barrier: patient expectations for communication

#### Suggested solution(s)

Patients will likely expect proactive communication about this substitution from their physicians such as letters, emails, or in-person conversation. Participants warned that skipping patient education and outreach, a critical step in effective communication, will likely lead to longer patient visits, greater patient confusion, and mistrust of the reasons for the change (i.e., the organization is just trying to save money).

### Barrier: clinician resistance

#### Suggested solution(s)

Provide education by presenting the evidence concisely in multiple formats (e.g., webinar, departmental meetings, email communication from leadership) prior to the rollout. A key focus of these materials should be on developing the clinician knowledge base, acceptance, and buy-in of the current guidelines.

### Barrier: clinician learning curve for follow-up algorithm

#### Suggested solution(s)

Developing clear data visualizations of the new primary HPV testing follow-up algorithm to address clinicians’ and administrators’ questions (i.e., a pocket card or simple one-page flowsheet). Department administrators suggested nurses in particular respond to visual resources (e.g., visualizations of workflow).

### Barrier: female nurses and physicians may have heightened resistance

#### Suggested solution(s)

Participants recommended remaining sensitive to the fact that many nurses and physicians are female and may have their own concerns or preconceptions about de-implementing the Pap test. Thus, it may be important to expend additional effort to gain buy-in from these clinicians.

Our findings can be applied to other health systems/settings considering implementation, particularly those within the USA or with a similar healthcare model (multiple payers, competition, concerns about patient loyalty, etc.). Studies from other countries that have implemented, or are currently implementing, primary HPV screening focus heavily on cost-effectiveness for national payers, cancer risks, and modeling downstream effects (e.g., potential increased colposcopy) and less on the concerns of clinician or patient buy-in and process change [11, 12, 51, 52]. Even in countries with a national health system organized around evidence-based care, there may be patient or clinician concerns about the change that our findings could help to address (e.g., patient concerns regarding unfamiliarity of primary HPV testing). Within the USA, there is the added layer of complexity that US guidelines currently recommend a suite of screening strategies with the new addition of primary HPV as an option (screening every 3 years with cytology alone, every 5 years with HPV testing alone, or every 5 years with HPV testing in combination with cytology (co-testing) in women 30–65 years, based on risk level) and European guidelines unequivocally recommend primary HPV screening [8]. Recommending multiple options for screening may compound the difficulties of implementation in the US context.

There are limitations to our findings. We used exploratory qualitative research methods to elicit participants’ perspectives. Consistent with this research approach, our study represents a small, but adequate sample size. While the findings may not be generalizable to all cervical cancer screening stakeholders within the KPSC region, our findings offer rich, context-specific insight into the experiences and perspectives of our clinical, administrator, and patient participants. We used standard and structured coding methods to extract themes from the raw data and employed independent coders to enhance analytic rigor.

## Conclusions

Practice substitution to replace an entrenched clinical practice with a new alternative is complex, involving both de-implementation and implementation processes. Leveraging available facilitators is key to ease the process for clinical and administrative stakeholders—in this case, emphasizing the efficiency of going from two tests to one. Identifying and proactively addressing clinical and patient fears regarding perceptions of cost-cutting and poorer quality of care is critical and unique to this type of practice change. As with other efforts to facilitate practice change, multicomponent and multilevel strategies for engagement, education, and sustainment will be required.

## Supplementary Information

**Additional file 1.** COREQ (COnsolidated criteria for REporting Qualitative research) Checklist.

**Additional file 2.** Implementing Primary HPV Testing Patient Interview Guide.

**Additional file 3.** Implementing Primary HPV Testing Physicians, Nurses, and Administrators Interview Guide.

**Additional file 4.** List of Potential Barriers – Primary HPV Testing for Routine Cervical Cancer Screening.

## Data Availability

No datasets are available from this study owing to the consents given by participants, which limits data to the research team only.
