# Supplementary material for: De-implementation and substitution of clinical care processes: stakeholder perspectives on the transition to primary human papillomavirus (HPV) testing for cervical cancer screening
Source: Implement Sci Commun. 2021 Sep 23;2:108. doi: 10.1186/s43058-021-00211-z (PMC8461958; doi:10.1186/s43058-021-00211-z)
Supplement: Supplementary file 2 — Additional file 2. Implementing Primary HPV Testing Patient Interview Guide. [file 43058_2021_211_MOESM2_ESM.pdf]

Implementing Primary HPV Testing
Patient Interview Guide

Interviewer instructions

Thank you so much for participating in this interview today. My name is _________ and I am a research associate/scientist with the Kaiser Permanente Southern California's Department of Research and Evaluation. I'm conducting this interview for a research study funded by the Patient-Centered Outcomes Research Institute.

Your participation in this interview is completely voluntary. You do not have to answer any questions that you do not want to answer, and you are free to stop participation at any time. Everything you say will remain confidential, and it will be summarized and reported anonymously.

The study is focused on understanding a potential new approach to cervical cancer screening. KPSC is currently using co-testing, which uses a Pap test and an HPV test to screen most women for cervical cancer. However, national organizations are now recommending a new approach for cervical cancer screening, known as the primary HPV testing. HPV is a virus that causes cervical cancer.

If KPSC were to adopt primary HPV testing for cervical cancer screening, it would result in a couple of practice changes for patients. For a primary HPV test, medical staff test for HPV infection by collecting a sample from the cervix, exactly as is done with a Pap test. The patient experience of the test procedure would be the same as the old approach. The difference between the Pap test and primary HPV testing lies in what is being examined from the collection of cells. One important change would be that not every woman would receive a Pap test under this new screening protocol. Those who have a normal test result for HPV would not receive a Pap test. Furthermore, women would receive information on the type of HPV if they have an HPV infection. Women would therefore be informed if they have the two types of HPV that carry the highest risk for cancer. Currently, women are only informed whether they have any high-risk HPV. The frequency of recommended screening under this new approach would not change.

Our study team believes that one of the best ways to help KPSC consider and prepare for a possible change to cervical cancer screening is to get input from members like you. We would therefore like to focus today's discussion on the challenges you think women might face accepting this new type of cervical cancer screening if it were adopted by our region. We would also like to get your ideas on strategies that the KPSC system or its doctors could use to address the challenges women might face with the new test. We will use the thoughts and opinions you share with us today to help create a summarized guidance document KPSC can use to consider whether to adopt a new test for cervical cancer screening.

We value your views on this topic and hope you will feel free to share your opinions. There are no right or wrong answers in this discussion. We will ask you to share your views, opinions, and experiences on a range of topics, so please feel free to be completely honest with your answers. Since we think it's important to capture your thoughts and opinions in your own words, we would also like to tape record the whole session. The tape recording and transcript of the discussion will remain completely confidential, will be stored on a secured server, and will only be used for research purposes.

Do you have any questions about the purpose of the interview or what to expect?

If you agree, I would like to begin the interview. I will turn on the audio-recorder now **[Interviewer Instructions: Confirm that the patient has reviewed and signed the audio-consent form prior to audio-taping the interview].**

Start Interview

SECTION I: GENERAL PERCEPTIONS OF GUIDELINES & MEDICAL EVIDENCE

Before we discuss the potential change to primary HPV testing for cervical cancer screening, we would like to spend the first few minutes discussing your overall views about medical evidence and guidelines that physicians typically rely on when providing care to their patients.

1. You may often hear terms like “medical guidelines” or “medical evidence” in the media or when you are seeking care from your physician. In general, what do you think when you hear these terms?
   Probes:
   a. What do medical guidelines or recommendations mean to you in thinking about your own health care?
   b. What do you know about medical guidelines in general?
   c. To what extent do you tend to rely on “medical evidence” or guidelines when thinking about your own health care needs and decisions?
2. Imagine you are going in for a visit to your physician expecting to complete what you believe to be a routine, standard medical test or procedure. During the visit, the physician informs you that they are making a change to this practice based on new medical guidelines or the current medical evidence (for instance, they are no longer performing test A, but switching to test B or maybe they are phasing out this type of routine screening test altogether). How would you likely react in this situation with your physician?
3. What level of “evidence” do you need or expect from your physicians to help you feel comfortable with decisions that are being made about your healthcare?
   Probes:
   a. What is the best way for physicians to approach talking to their patients about medical guidelines or evidence?
   b. When talking to patients about new guidelines or evidence, what specific types of resources should be offered to patients to help them understand these changes?
   c. How much detail or level of evidence should a provider share with patients about changing recommendations so that patients are more likely to believe or value the quality of the evidence?

SECTION II: BARRIERS TO ACCEPTANCE OF PRIMARY HPV TEST

Great, thank you for your thoughtful responses. Now we would like to hear your thoughts regarding concerns women may have about KPSC’s potential transition to using the primary HPV test for cervical cancer screening.

1. What is your reaction to hearing that KPSC is considering adopting this new approach to cervical cancer screening, the primary HPV test?
   Probes:
   a. Emotional response
   b. Types of questions this raises for the participant
      i. Why is KPSC considering the switch?
   c. How do you think other women who are KPSC members would react if their doctor told them KPSC would be switching to this new approach?

2. What parts of the new approach and potential changes to cervical cancer screening concern you at this time?

3. What types of concerns, if any, would you have about getting tested for HPV to screen for cervical cancer?
   Probe for underlying reasons for concerns:
   a. Knowledge/attitudes related to preventive care
   b. Knowledge/attitudes related to cervical cancer screening
   c. Knowledge of HPV, link between HPV and cervical cancer
   d. Anxiety/fears around testing
   e. Lack of agreement or comfort with HPV testing
   f. Stigma associated with HPV testing
   g. Anxiety level after positive HPV test result
   h. Stigma associated with positive HPV test result

4. Do you think some types of patients would have more concerns than others about these changes to cervical cancer screening? If so, please describe.
   Probes:
   a. Limited English-proficiency patients
   b. Patients from different cultural groups
   c. Pregnant patients
   d. Older versus younger patients

5. Of all the issues just discussed, which would you rank as the most urgent for KPSC to address assuming the region decided to adopt this change to cervical cancer screening?

SECTION III: STRATEGIES FOR INCREASING ACCEPTANCE OF PRIMARY HPV TEST

Now we would like to switch to hearing your thoughts on strategies KPSC can use to address the concerns you have identified.

1. If KPSC made the switch to primary HPV testing for cervical cancer screening in the future, how could your doctors help address your concerns about the new screening approach when it was rolled out?

   Probes:

   a. Type of information shared – why KPSC is doing the switch
   b. Provider handling of patient questions – feeling that concerns and/or questions about the new test are addressed
   c. Provider communication style
   d. Patient satisfaction with the cervical cancer screening experience
   e. Type of scientific evidence shared by provider, and how communicated

2. What do you think KPSC could do to address your concerns about the potential new screening approach?
   a. Patient education - type and timing of information shared – why KPSC is doing the switch?
   b. Type and format of system-level communication of results
   c. Patient satisfaction with the cervical cancer screening experience
   d. Type of scientific evidence shared by health system, and how communicated

3. Of all the strategies just discussed, which would you rank as the most urgent for KPSC to start doing if it adopted this new approach to cervical cancer screening?

SECTION IV: CONCLUSION

That basically concludes my questions for today, unless you have any final thoughts or suggestions.

1. Is there anything important you would like to bring up that you feel we haven't covered today?

End of Interview Questions.

That concludes my questions for today, unless you have any final thoughts or suggestions. Again, I want to thank you very much for taking the time to participate in this interview.
