# Supplementary material for: De-implementation and substitution of clinical care processes: stakeholder perspectives on the transition to primary human papillomavirus (HPV) testing for cervical cancer screening
Source: Implement Sci Commun. 2021 Sep 23;2:108. doi: 10.1186/s43058-021-00211-z (PMC8461958; doi:10.1186/s43058-021-00211-z)
Supplement: Supplementary file 3 — Additional file 3. Implementing Primary HPV Testing Physicians, Nurses, and Administrators Interview Guide. [file 43058_2021_211_MOESM3_ESM.pdf]

Implementing Primary HPV Testing

Physicians, Nurses, and Administrators Interview Guide

Interviewer instruction: *Provide copy of Study Information Sheet to participant.*

Hello, my name is [________] and I will be talking with you today. We are conducting interviews with KPSC clinicians and teams (physicians, nurses, and department administrators) to better understand factors that influence the implementation of a routine cervical cancer screening. Current guidelines from national organizations now recommend a new approach to cervical cancer screening. We are interested in your views including your perceptions of potential barriers and facilitators to implementation and your medical centers' readiness to change if a new approach to cervical cancer screening is adopted. Additionally, we will ask you to share any suggestions you have for ways to overcome identified challenges to implementation at the local level.

Your participation in this interview is extremely valuable and appreciated. Please note:

- The interview is expected to last approximately 30-45 minutes.
- We would like to record the interview so that we are able to capture the discussion in full, as well as how you say things (e.g., to capture points that you particularly emphasized).
- <u>This interview is not a test of your knowledge and we will not focus on any one person's answers or practices, but rather use the information to better understand comparative strategies for implementation across our region for routine cervical cancer screening.</u>

Do you have any questions about the purpose of the interview or what to expect?

If you agree, I would like to begin the interview. I will turn on the audio-recorder now.

Start Interview

Section 1: Awareness, Knowledge & Beliefs (Characteristics of Individuals)

1. Prior to today, what level of awareness did you have that KPSC is considering adopting a new approach to cervical cancer screening in 2019 – e.g. a change from the current practice of co-testing to primary HPV testing for routine cervical cancer screening?
    a. Probe(s):
        i. What is your initial reaction to this planned practice change?
        ii. Do you believe there is a strong need for this practice change across our region?
        iii. How does implementation of the intervention align with other organizational goals?

Section 2: Strength, Complexity, Design (Intervention Characteristics)

The next questions address challenges you believe your team may encounter if our region were to adopt a new approach to *cervical cancer screening, e.g.,, switching from co-testing to primary HPV screening,* ***as well as your suggestions for overcoming any identified barriers.***

1. In your opinion, what kind of supporting evidence or proof is needed about the effectiveness of primary HPV testing to get staff at your medical center on board?
    a. Probe: Do you believe the transition to primary HPV testing for cervical cancer screening will improve patient care, based on the current evidence?
2. Has your medical center previously implemented a comparable practice change?
    a. Probes:
        i. If so, what were some of the biggest challenges to that implementation process?
        ii. Strategies employed for overcoming those challenges?
3. How easy or difficult do you believe it will be for you and your team to transition from co-testing to primary HPV testing to routinely screen for cervical cancer?
    a. Probe: In your mind, what challenges would you expect to encounter in making this transition?

INTERVIEWER INTSRUCTIONS: INTRODUCE LIST OF POTENTIAL BARRIERS HANDOUT:

Here is a list of potential barriers organized by stakeholder assuming this potential practice change were to be implemented at KPSC (e.g., switch from co-testing to primary HPV screening). Please take a moment to review the list in the column and then highlight all of the barriers you believe will be challenging for your team during this transition using the yellow highlighter marker provided.

4. **[Interviewer will first briefly review and discuss selected barriers with participant]**
    a. Probe: Of the barriers you've highlighted, which do you believe is the most pressing barrier to this practice change within your setting?

5. Thinking about your clinic, how may this transition to primary HPV screening have to be tailored or adapted in order to work effectively in your setting?
    a. Probe: What is it about your setting that you think will make this type of tailoring or adaptation necessary during the transition?
6. What, if anything, would make it easier for you and your team to successfully shift from co-testing to primary HPV testing?
    a. Probe: What training resources are necessary to ensure that your team members will be adequately prepared to conduct primary HPV screening instead of co-testing for routine cervical cancer screening?

Section 3: Patients' Needs/Resources (Outer Setting)

Thank you so much for your responses thus far, they have been extremely helpful. Let's now spend a few minutes talking about patient reactions to this hypothetical practice change.

1. What patient-level barriers or concerns do you anticipate as the process of transitioning unfolds at your medical center?
    a. Probe: What suggestions do you have for addressing these challenges?
2. What types of reactions do your patients have when you raise the issue of HPV testing in general?
    a. Probe: How do you typically broach the subject of HPV testing with patients? What strategies do you use for this conversation?

Section 4: Networks, Culture, Climate & Readiness (Inner Setting)

We are also interested in learning more about the context of your specific setting, including team culture, clinical and administrative leadership, and resources.

1. How do you typically learn new information about initiatives like the practice change for cervical cancer screening KPSC is currently undergoing?
2. When you need to get something done or to solve a problem, who are your "go-to" people?
    a. Probe: Who on your team do you imagine will be most involved in rolling out this practice change at your medical center?
3. How would you describe the culture of your team?
    a. Probes:
        i. What is the first word/phrase that comes to mind when thinking about the culture of your team?
        ii. How much personal responsibility do you feel for improving clinical procedures/practices?
4. How well does the intervention fit with existing cervical cancer screening work processes and practices in your setting?
    a. Probes:
        i. Please describe how you believe the practice change will be adopted and integrated into your workflow?
        ii. What challenges do you perceive? Suggested solutions?

Let's now explore your perceptions of the degree to which your team and clinical/administrative leadership are likely to support this hypothetical practice change effort within your setting.

5. Do you expect to have sufficient buy-in from you team and leadership to implement the substitution of primary HPV testing for co-testing at your clinic?
6. Thinking about this current initiative, what specific steps do you think your senior leadership/clinical management will likely take to establish goals for the team?
    a. Probes:
        i. How will they likely measure those goals?
        ii. What methods do you think they will use to hold staff accountable for achieving results?

Section 5: Provider Self-Efficacy (Individual Characteristics)

1. How confident are you that you can switch to primary HPV testing in your own practice?
    a. Probe: How about your colleagues – how confident do you think they will feel?

Section 6: Identifying Champions-Key Leaders (Process)

1. Who are the key influential individuals on your team to get on board with this transition – we would define these people as likely champions willing to go above and beyond what might be expected during the planned transition to primary HPV testing?
2. Who would you say would be the leader of this effort in your clinic—it could be you, or someone else on your team?

End of Interview Questions.

That concludes my questions for today, unless you have any final thoughts or suggestions. Again, I want to thank you very much for taking the time to participate in this interview.
