# Supplementary material for: De-implementation and substitution of clinical care processes: stakeholder perspectives on the transition to primary human papillomavirus (HPV) testing for cervical cancer screening
Source: Implement Sci Commun. 2021 Sep 23;2:108. doi: 10.1186/s43058-021-00211-z (PMC8461958; doi:10.1186/s43058-021-00211-z)
Supplement: Supplementary file 4 — Additional file 4. List of Potential Barriers – Primary HPV Testing for Routine Cervical Cancer Screening. [file 43058_2021_211_MOESM4_ESM.pdf]

List of Potential Barriers – Primary HPV Testing for Routine Cervical Cancer Screening		
**Directions: Please place a check mark to the right of each item below if you agree that it is a potential barrier**		✓
**System-level**		
1	Inability to perform proper follow-up and treatment of women with positive results (i.e., time to colposcopy)	
2	Low sensitivity for cervical cytology [facilitator]	
3	Provider lack of time to properly educate patient about the change during an office visit	
4	Lab capacity for new test	
5	Unable to provide proper supply for the new test materials	
6	Others (please write in):	
**Provider-, Nurse- and Staff-level**		
1	Clinician/nurse awareness/knowledge of primary HPV screening	
2	Clinician/nurse lack of knowledge about rationale behind the guideline changes	
3	Clinician/nurse perceptions of primary HPV screening	
4	Clinician/nurse lack adequate training in HPV testing	
5	Provider belief that HPV testing is unsafe for pregnant women	
6	Provider/nurse knowledge of the follow-up algorithm	
7	Time needed to counsel/educate women about the new test before screening	
8	Time needed to counsel/education women after positive test result	
9	Unsure how to bring up new testing procedures with patients/patient-provider communication	
10	Clinician concern that “My patients don’t like change.”	
11	Uptick in questions/number of emails received through KP.org during transition	
12	Concerns about patient adherence to screening	
13	Concerns about patient satisfaction (with transition process)	
14	Nursing teams’ proficiency in preparing for the new screening exam	
15	Concern about overwhelming the colposcopy clinic	
16	Workflow challenges for medical staff/nursing staff	
17	Patients’ willingness to return for colposcopy	
18	Patients’ willingness to return for repeated testing	
19	Others? (please write in)	
**Patient-level**		
1	Patients unaware of changes in cervical cancer screening guidelines	
2	Patients’ knowledge of HPV testing and why the switch?	
3	Pap test preference/satisfaction with the cervical cancer screening experience (status quo)	
4	Adverse attitude toward change	
5	Fear of results/anxiety level after positive HPV test result	
6	Stigma of HPV-positive results	
7	Stigma or shame of getting HPV test	
8	Disagreement with HPV test itself	
9	Discomfort of getting an STD screening to replace PAP test	
10	Patient perception that KPSC is just trying to save money by taking away PAP test	

11	Unsure of purpose of PAP test and/or HPV test	
12	Feeling disempowered/loss of control re: her own preventive care	
13	Distrust in doctor or system	
14	Others (please write in?)	
